# The Disulfide Bonds in Glycoprotein E2 of Hepatitis C Virus Reveal the Tertiary Organization of the Molecule

**DOI:** 10.1371/journal.ppat.1000762

**Published:** 2010-02-19

**Authors:** Thomas Krey, Jacques d'Alayer, Carlos M. Kikuti, Aure Saulnier, Laurence Damier-Piolle, Isabelle Petitpas, Daniel X. Johansson, Rajiv G. Tawar, Bruno Baron, Bruno Robert, Patrick England, Mats A. A. Persson, Annette Martin, Félix A. Rey

**Affiliations:** 1 Institut Pasteur, CNRS URA3015, Unité de Virologie Structurale, Paris, France; 2 Institut Pasteur, Plate-forme d'Analyse et de Microséquençage des Protéines, Paris, France; 3 Institut Pasteur, CNRS URA3015, Unité de Génétique Moléculaire des Virus à ARN, Paris, France; 4 CNRS UPR3296, Laboratoire de Virologie Moléculaire et Structurale, Gif-sur-Yvette, France; 5 Karolinska University Hospital, Department of Medicine, Center for Molecular Medicine, Stockholm, Sweden; 6 Institut Pasteur, CNRS URA2185, Plate-forme de Biophysique des Macromolécules et de leurs Interactions, Paris, France; 7 CEA, CNRS URA2096, Institut de Biologie et de Technologie de Saclay, Gif-sur-Yvette, France; The Rockefeller University, United States of America

## Abstract

Hepatitis C virus (HCV), a major cause of chronic liver disease in humans, is the focus of intense research efforts worldwide. Yet structural data on the viral envelope glycoproteins E1 and E2 are scarce, in spite of their essential role in the viral life cycle. To obtain more information, we developed an efficient production system of recombinant E2 ectodomain (E2e), truncated immediately upstream its trans-membrane (TM) region, using *Drosophila melanogaster* cells. This system yields a majority of monomeric protein, which can be readily separated chromatographically from contaminating disulfide-linked aggregates. The isolated monomeric E2e reacts with a number of conformation-sensitive monoclonal antibodies, binds the soluble CD81 large external loop and efficiently inhibits infection of Huh7.5 cells by infectious HCV particles (HCVcc) in a dose-dependent manner, suggesting that it adopts a native conformation. These properties of E2e led us to experimentally determine the connectivity of its 9 disulfide bonds, which are strictly conserved across HCV genotypes. Furthermore, circular dichroism combined with infrared spectroscopy analyses revealed the secondary structure contents of E2e, indicating in particular about 28% β-sheet, in agreement with the consensus secondary structure predictions. The disulfide connectivity pattern, together with data on the CD81 binding site and reported E2 deletion mutants, enabled the threading of the E2e polypeptide chain onto the structural template of class II fusion proteins of related flavi- and alphaviruses. The resulting model of the tertiary organization of E2 gives key information on the antigenicity determinants of the virus, maps the receptor binding site to the interface of domains I and III, and provides insight into the nature of a putative fusogenic conformational change.

## Author Summary

Little is known about the structure of the envelope glycoproteins of the hepatitis C virus (HCV), in spite of their essential role in the viral cycle of this major human pathogen. Here, we determined the connectivity of the 9 disulfide bonds formed by the strictly conserved 18 cysteines of the ectodomain of HCV glycoprotein E2. We show that this information, together with important functional data available in the literature, impose important restrictions to the possible three-dimensional fold of the molecule. Indeed, these constraints allow the unambiguous threading of the predicted secondary structure elements along the polypeptide chain onto the template provided by the crystal structures of related flavi- and alphavirus class II fusion proteins. The resulting model of the tertiary organization of E2 shows the amino acid distribution among the characteristic class II domains, places the CD81 binding site at the interface of domains I and III, and highlights the location of a candidate fusion loop.

## Introduction

The hepatitis C virus (HCV) is a major cause of chronic liver disease worldwide, leading to cirrhosis and hepatocellular carcinoma [1]. In spite of being the focus of intense research efforts, no vaccine is available against HCV, and current therapeutic treatments have limited efficacy and significant side effects [2]. HCV belongs to the *Flaviviridae* family of enveloped, positive-strand RNA viruses [3]. Structural studies on this virus are difficult, in part because it propagates poorly in cell culture, and particles isolated from infected patients are heterogeneous and not amenable to a detailed structural characterization. Little structural information is available on the envelope proteins, which are heavily glycosylated, display hypervariable loops, and are stabilized by numerous disulfide bridges [4]. The folding kinetics of these proteins are slow, requiring several hours for completion of a complex process involving various ER chaperones of the infected cell [5]. These properties make their recombinant production - in a native conformation and in sufficient amounts for structural studies - a difficult endeavor. Yet structural information on the HCV envelope proteins would be extremely valuable, given that they carry the main antigenic determinants of the virus and play an essential role in cell entry by binding to specific receptors and inducing membrane fusion. HCV has indeed been shown to depend on a number of cellular molecules for entry, including CD81 [6] and the tight junction transmembrane proteins claudin 1, 6, 9 and occludin [7]–[9], as well as the scavenger receptor B1 (SR-B1) [10]. The LDL receptor also plays a role in HCV uptake, in line with the observation that HCV particles in infected plasma are associated with LDL species [11]. A direct interaction of the HCV envelope protein E2 with CD81 and SR-B1 has been demonstrated, and these interactions were shown to be necessary but not sufficient for cell entry. The mode of interaction of HCV with the claudins and occludin is not understood at present.

The HCV genome codes for a single polyprotein precursor about 3000 amino acids long, spanning the ER membrane multiple times. It contains, sequentially, the viral proteins in the order Nter-C-E1-E2-p7-NS2-NS3-NS4A/B-NS5A/B-Cter. The N-terminal 1/4^th^ of the precursor corresponds to the structural proteins C (Core), E1 and E2 (envelope proteins 1 and 2) and p7, which functions as a proton channel. The remainder of the polyprotein contains the non-structural (NS) proteins, which have enzymatic and other activities that are necessary for virus replication. The mature viral proteins are generated by proteolytic processing of the precursor by cellular and viral proteases [3]. In particular, the envelope proteins are generated by host-cell signalases. E1 and E2 are type 1 trans-membrane (TM) proteins with a large N-terminal ectodomain and almost no cytoplasmic tail. In the best characterized HCV strain H77, E1 and E2 are 192 and 366 amino acids long and contain 6 and 11 potential N-linked glycosylation sites, respectively. Biochemical studies have shown that E1 and E2 fold as a heterodimer, which is found at the surface of viral particles and is thought to be the functional glycoprotein form [4]. There are currently 6 identified HCV genotypes further divided into several subtypes [12]. The amino acid sequence identity between envelope proteins from different genotypes is about 68% for the most distant genotypes. E2 has been shown to contain 3 hypervariable regions that can be deleted without affecting the overall fold of the protein, as assayed by binding to conformation-sensitive mAbs and CD81 [13]–[15].

The genomic organization of HCV is characteristic of all members of the *Flaviviridae* family [3]. In particular, the envelope proteins are present in tandem within the polyprotein precursor. This arrangement of the structural part of the genome is characteristic of viruses encoding class II fusion proteins, reviewed in [16]. These proteins have been extensively characterized, structurally and biochemically, for viruses in the flavivirus genus within the *Flaviviridae* family [17]. Class II proteins have a common tertiary structure, which has also been observed in the fusion protein of Semliki Forest virus (SFV), an alphavirus belonging to a separate family of enveloped, positive-strand RNA viruses, the *Togaviridae* [18]. *Togaviridae* and *Flaviviridae* display the same gene order in the structural part of their genomes. There is no amino acid sequence similarity in the alpha- and flavivirus fusion proteins, however, and in spite of sharing a common fold, they are stabilized by a different pattern of disulfide bonds. Viruses within the *Flaviviridae* families have no sequence similarity across the various genera either, and the fusion proteins from each genus also appear to have their own characteristic pattern of disulfide bonds. Yet the conservation of the class II fold across viral families in the absence of sequence conservation strongly suggests that it is also conserved across the different genera within the respective families.

A further feature of class II viral fusion proteins is that they fold as a heterodimer with the upstream glycoprotein in the polyprotein precursor. This heterodimer later dissociates to drive membrane fusion upon interactions with the host cell. The first glycoprotein in the tandem thus acts as chaperone for folding the second one, which has the membrane fusion role. The chaperone function was experimentally demonstrated for the flavivirus prM [19] and the alphavirus p62 [20] glycoproteins, which precede the fusion proteins E and E1, respectively, in the precursor polyprotein. The effect on folding appears to be reciprocal, since both p62 and prM also adopt their native conformation only in presence of the respective accompanying fusion protein (unpublished observations). Importantly, heterodimerization upon folding has also been characterized for viruses belonging to other genera in the two families, and in particular for HCV [5],[21].

Flavivirus E and alphavirus E1 change into a homotrimer upon interaction with lipids in the acidic environment of a target cell endosome, in a process that drives fusion of the viral and endosomal membrane and results in infection of the cell [22],[23]. This process involves homodimer (E-E, flavivirus) or heterodimer (E2-E1, alphavirus) dissociation, followed by homotrimerization of E (flavivirus) or E1 (alphavirus) upon binding to lipids. The tertiary structure of class II viral fusion proteins contains predominantly β-sheets segregated into three distinct domains arranged linearly, resulting in a rod-like molecule. The central domain 1 (DI) is a β-sandwich with two long insertions in loops connecting adjacent β-strands. These insertions form an elongated “fusion” domain (DII), carrying the “fusion loop” in the first of the two insertions, at the distal end of the rod. The fusion loop is a segment of the polypeptide chain that inserts into the target membrane in the first step of membrane fusion. At its C-terminal end, DI is connected via a flexible linker to domain 3 (DIII), which is located at the opposite side with respect to DII, giving rise to the linear organization of the molecule. DIII plays an important role in the fusogenic conformational change, during which it relocates to the side of the molecule, resulting in the characteristic “hairpin” conformation of the protein, which drives membrane fusion. This relocation involves a considerable stretching of the segment connecting DI to DIII, the region that changes most dramatically in conformation during the fusogenic transition (reviewed in [16]).

Although there is no direct experimental evidence demonstrating the role of E2 as the HCV fusion protein, the compelling similarities to viruses with class II fusion proteins suggest that membrane fusion is at least one of its biological roles. It is worth noting, however, that while totally unrelated viruses can have structurally homologous fusion proteins (for example, rhabdoviruses, herpesviruses and baculoviruses, reviewed in [24]), related viruses can use non-homologous fusion proteins, as is the case with paramyxoviruses and rhabdoviruses, which belong to the *Mononegavirales* order (reviewed in [25]). Yet the fact that viruses belonging to different genera in the *Flaviviridae* and *Togaviridae* families display a genomic arrangement that is the signature of class II fusion proteins, together with the additional common features outlined above, makes it very likely that they code for envelope glycoproteins that are at least distantly related to class II proteins.

A model for E2 has actually been proposed based on the structure of the flavivirus E protein homodimer [26], although no evidence is available for homodimerization of HCV E2, which forms a heterodimer with E1 in infectious virions [4]. More importantly, this model does not take into account the location of the strictly conserved cysteine residues forming 9 disulfide bonds [27]. This model also lacks the third domain, which is important in the fusogenic transition. Moreover, it was also proposed that the membrane fusion function could be carried by HCV glycoprotein E1 (i.e., the first glycoprotein in the tandem) [28],[29], in spite of the similarities with flavi- and alphaviruses discussed above, and in the absence of experimental support. Furthermore, a bioinformatics model for HCV E1 as a truncated class II protein was reported [30], postulating that E1 has the fold of DII of an alpha- or flavivirus fusion protein, but neglecting the fact that in class II proteins, DII works in conjunction with the other two domains covalently linked within the polypeptide to induce membrane fusion. The corollary is that controversial hypotheses have been reported concerning the identity of the HCV fusion protein. It is therefore important to stress that the structural studies performed over the years on viral membrane fusion proteins strongly suggest that most animal enveloped viruses encode fusion proteins belonging to one of the three currently characterized structural classes [31]. It is thus highly unlikely that HCV would have acquired a totally novel fusion machinery (for instance, one in which E1 would be the membrane fusion protein), especially when taking into account the similarities to class II proteins presented above.

**Figure 1:**
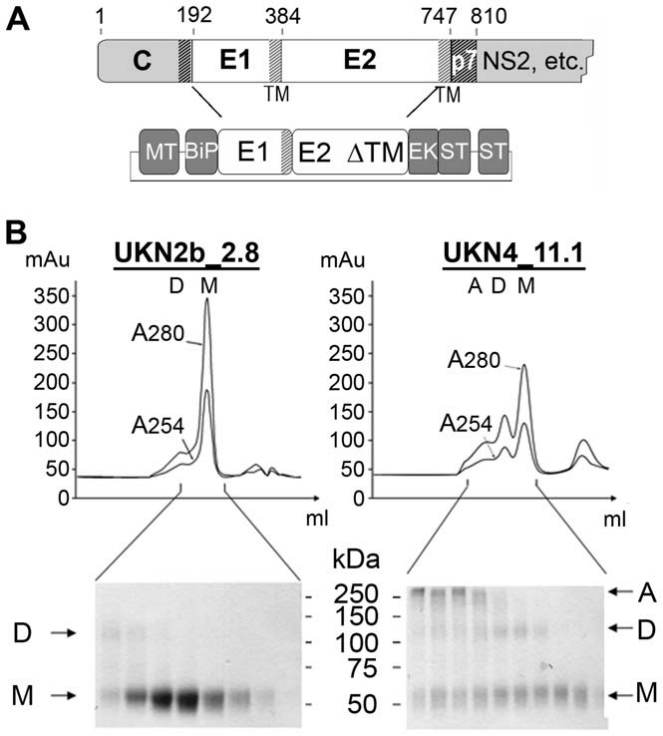
Production and biochemical characterization of HCV E2e. A) Schematic diagram of the HCV genome region coding for the structural proteins and the constructs used to make stable S2 cell transfectants expressing E2e. MT: inducible metallothionin promoter, BiP: *Drosophila* BiP Signal peptide, EK: enterokinase cleavage site, ST: Strep-Tag. B) Elution profile of E2e from the HCV isolates indicated from an Sdx200 size exclusion column. Bottom panels: Non-reducing SDS-PAGE analysis of the eluted fractions. Arrows indicate multimeric (A), dimeric (D) and monomeric (M) forms of the protein.

In order to bring more insight into the tertiary structure of HCV E2, we report here the experimental identification of the connectivity of the 9 disulfide bonds present in the recombinant E2 ectodomain (E2e) generated by expression of the E1-E2ΔTM portion of the HCV genome in *Drosophila* S2 cells (Fig. 1A). The absence of the transmembrane (TM) segment in E2 leads to secretion of its ectodomain after folding in the presence of E1. This approach is based on previous results leading to production of recombinant dengue virus E protein in the presence of its viral chaperone prM [32]. We tested the conformation of recombinant HCV E2e biochemically and functionally, showing that it reacts with conformation-sensitive antibodies and inhibits infection of Huh7.5 cells by infectious HCV particles (HCVcc) in a dose-dependent manner. Knowledge of the disulfide bonds, along with functional data on deletion mutants [14] and CD81 binding [26],[33],[34], together with secondary structure predictions, provide sufficient constraints to reconstitute the tertiary organization of the molecule. This information allowed the threading of the E2e polypeptide chain onto a class II template by matching the predicted β-strands. The resulting model reveals the distribution of the amino acids of HCV E2 among the different domains, maps the CD81 binding site to the DI/DIII interface, and highlights a strictly conserved segment of the polypeptide chain as a strong candidate for the HCV fusion loop.

## Results/Discussion

### Biochemical characterization of recombinant E2e

We generated stable *Drosophila* S2 cell-lines expressing the E1-E2ΔTM segment of the precursor polyprotein (Fig. 1A) from 9 isolates spanning all 6 HCV genotypes and 4 subtypes (Table 1). In order to ensure that the recombinant E2 proteins were functional, we selected isolates previously tested for entry of retroviral particles pseudotyped with HCV glycoproteins (HCVpp) with the corresponding sequences [35]. Induction of expression at high cell density with CdCl_2_ resulted in accumulation of relatively high levels of secreted E2e in the cell culture medium. We purified the protein to homogeneity from the supernatant (described in Text S1), with the yields listed in Table 1. E2e from the different isolates behaved similarly, as judged by size exclusion chromatography (SEC) followed by SDS-PAGE analysis under reducing and non-reducing conditions and Coomassie blue staining (Fig. 1). In a typical SEC profile, the majority of the protein elutes at a volume corresponding to a monomer, with additional minor peaks corresponding to disulfide linked dimers and higher multimers, which vary depending on the construct analyzed. The monomeric form was efficiently separated from the other species by pooling the corresponding fractions. Analytical ultracentrifugation and small angle X-ray scattering confirmed the monomeric state of the protein eluted in these fractions (data not shown). Once isolated, E2e from all constructs listed in Table 1 remained monomeric and showed no tendency to associate into disulfide-linked aggregates over time. The construct corresponding to the genotype 2b isolate (UKN2b_2.8) reproducibly yielded the highest amounts of purified monomeric protein (Table 1). The construct from genotype 4 (UKN4_11.1 isolate) yielded a significant fraction of disulfide-linked aggregates (Fig. 1), which are likely to correspond to misfolded protein. E2e from the remaining 7 constructs yielded slightly lower yields of purified, monomeric protein than did the genotype 2b construct, the lowest yields being from the gentoype 6 isolate (Table 1). The SEC profiles from the 7 other constructs were intermediate between the two chromatograms shown in Fig. 1.

**Table 1:** Production yields of E2e constructs from the 9 selected HCV isolates.

Strain/Isolate	Genotype	Genbank Accession	Average Yields *
H77	1a	GI:130461	230 µg/l
Con1	1b	GI:5420377	180 µg/l
JFH-1	2a	GI:116078059	200 µg/l
J6	2a	GI:221651	150 µg/l
UKN2b_2.8	2b	GI:58198335	300 µg/l
UKN3a_1.28	3a	GI:58198337	200 µg/l
UKN4_11.1	4	GI:58198341	190 µg/l
UKN5_14.4	5	GI:58220848	220 µg/l
UKN6_5.340	6	GI:58220846	70 µg/l

*The yields specified correspond to the purified monomeric form of E2e. Significant differences in production yields (±20%) were observed between independent preparations.

### Conformational characterization

Because of the higher production yields, we pursued most of the biochemical characterization using E2e from genotype 2b, to which we will refer to as E2e in the rest of the manuscript, except when explicitly stated. Yet because the best functionally characterized HCV strain is H77 (genotype 1a), we use the amino acid numbering corresponding to the H77 polyprotein throughout the manuscript.

**Figure 2:**
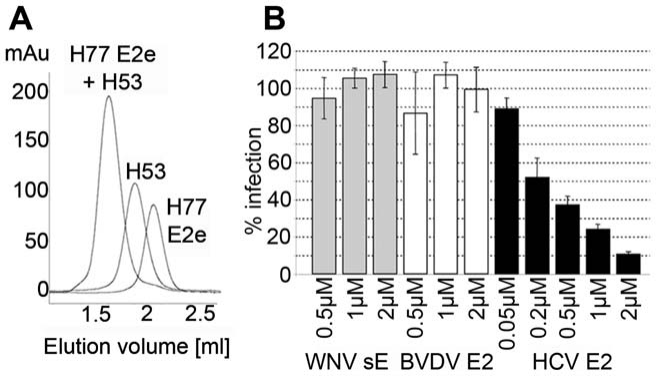
Functional and conformational characterization of HCV E2e. A) Stoichiometric complex formation between H77 E2e and mAb H53. H77-E2e, mAb H53 and a mixture of the two (molar ratio 2∶1) were loaded to the column (in three different runs) (E2e∼50kD, H53∼150kD, complex∼250kD). No peaks corresponding to either of the isolated proteins were observed in the profile of the complex, indicating a 2∶1 complex stoichiometry and a high affinity of H77 E2e for mAb H53. B) Dose-dependent inhibition of infection of Huh7.5 cells by HCVcc. Huh-7.5 cells were preincubated with increasing concentrations of HCV E2e, WNV sE or BVDV E2e and subsequently infected with HCVcc in the corresponding recombinant protein concentration. The number of infected foci was determined after immunofluorescence analysis detecting intracellular HCV core antigen. The columns represent mean values of duplicates in a representative experiment; bars indicate mean deviation, 100% corresponds to the mean value of the infection in the presence of the control proteins.

Pull-down assays showed that E2e efficiently binds the CD81 large external loop (LEL), as well as conformation-sensitive mAbs CBH-4B and CBH-4D [36] (Fig. S1A). To further confirm that CD81 and the conformation-sensitive mAbs bind stoichiometrically to monomeric E2e, we used SEC to analyze the formation of various E2e/ligand complexes in defined ratios. The resulting chromatograms display a quantitative shift of the peak from monomeric protein to an E2e/mAb complex with a 2∶1 stoichiometry, as expected (Figs. 2A and S1B). The SEC profile displayed in Fig. 2 shows the well-characterized conformation-sensitive mAb H53 that is specific for genotype 1a, whereas Fig. S1B shows the same analysis of E2e from the genotype 2b isolate and the human conformation-sensitive mAb CBH-4D. Similarly, SEC analysis using the Fab fragment of the corresponding mAbs under the same conditions, yielded a 1∶1 E2e/Fab stoichiometry, as expected (data not shown). SEC analysis revealed that E2e also forms a stoichiometric complex with the CD81 LEL (data not shown). The monomeric fraction from all isolates listed in Table 1 yielded similar results - except perhaps for the genotype 6 isolate, which was not tested - strongly suggesting that recombinant E2e adopts a conformation closely resembling that of authentic E2 present on virions.

We further tested the ability of E2e to compete with infectious HCV particles for entry receptors, by measuring its ability to inhibit infection of Huh-7.5 cells by HCVcc (Fig. 2B). As a control, we tested in parallel the effect of the flavivirus E protein ectodomain (sE) from West Nile encephalitis virus (WNV), as well as the ectodomain of pestivirus E2 (pE2e) from the bovine viral diarrhea virus (BVDV) produced under identical conditions. In contrast to the control proteins, HCV E2e exerted a clear dose-dependent inhibition of the infection. At the lowest concentration tested (0.05 µM), 10% inhibition was observed, which increased with protein concentration to reach 90% inhibition at 2 µM of HCV E2e. This effect is in line with the observation that E2e makes a stoichiometric complex with CD81, as described above.

### Secondary structure analyses

**Figure 3:**
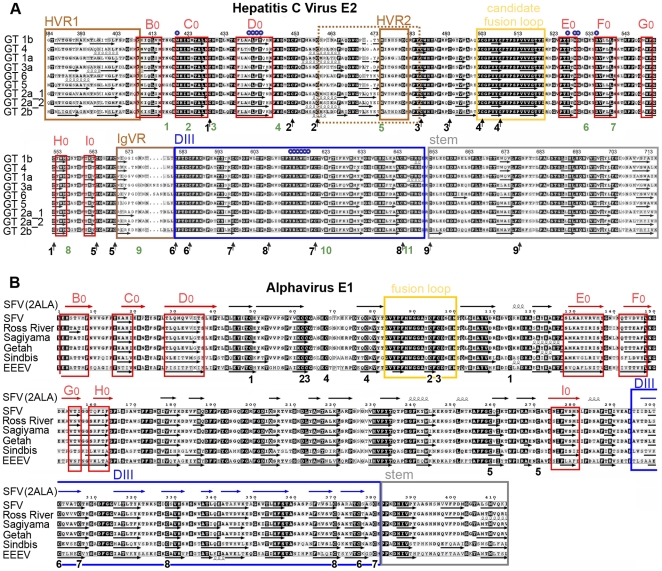
Amino acid sequence alignments and secondary structure predictions. The secondary structure of HCV E2e and alphavirus sE1 was predicted using the program DSC [48], which was selected because the predictions matched more closely the crystallographically determined secondary structure elements of class II proteins. Arrows or spirals under each sequence indicate predicted β-strands or α-helices, respectively. A) The main elements of the tertiary structure model of HCV E2 are framed: assigned strands in DI (red, labeled) and DIII (blue), putative fusion loop (yellow), the stem (grey) and regions that can be deleted without affecting the protein conformation (brown). The 18 cysteines, which form the 9 disulfides, are marked with arrows and numbered according to the disulfide bond (Table 2) under the sequences. N-linked glycosylation sites are numbered in green. Residues known to interact with CD81 are marked with small blue circles. The numbering corresponds to the HCV H77 polyprotein. B) Comparison with a class II fusion protein of known structure. The experimentally determined secondary structure of SFV E1 taken from the crystal structure (PDB 2ALA, [49]), shown with symbols (arrows or spirals) above the sequence alignment, was compared with secondary structure predictions for the E1 ectodomain of selected alphaviruses. Experimentally determined β-strands in DI, DII and DIII are colored red, black and blue, respectively. The red frames indicate the consensus predicted strands in DI, for easier comparison with panel A. Similarly the fusion loop (yellow), the region corresponding to the crystallographically identified DIII (blue), and the stem (grey) are framed. The 16 cysteines forming the 8 disulfides are numbered in black according to the disulfide bond under the sequences. The numbering starts with the first amino acid of the E1 glycoprotein.

**Figure 4:**
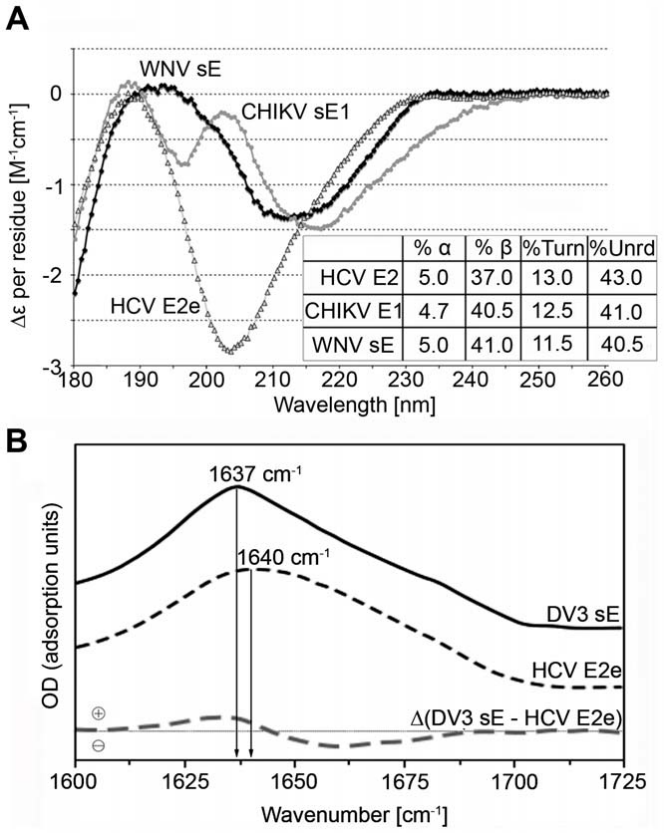
Experimental analysis of the secondary structure of HCV E2e. A) Far-UV CD spectra obtained with recombinant HCV E2e (empty triangles) and the controls CHIKV sE1 (grey circles) and WNV sE (black diamonds). The inset shows the estimated fraction of α-helix, β-sheet, turns and unordered polypeptide chain for the 3 proteins. B) FTIR spectra of HCV E2e (dashed black line) and DV3 sE (solid black line) and the difference spectrum of the two after normalization (dashed grey line) in the amide I band region.

Computer algorithms for secondary structure prediction using amino acid alignments of E2 from all 6 HCV genotypes predict predominantly β-strands in E2e (Fig. 3), consistent with the fold of class II fusion proteins. We used recombinant E2e to experimentally analyze its secondary structure composition with two complementary methodologies, circular dichroism (CD), which is sensitive to the presence of α-helices, and Fourier transform infrared (FTIR) spectroscopy, which readily detects β-sheets present in a protein. We carried out these tests in parallel with recombinant control class II envelope proteins of known structure available in the laboratory. For the CD measurements, the controls were WNV sE, ([37], PDB 2I69) and the ectodomain of glycoprotein E1 (sE1) of Chikungunya virus (CHIKV), which displays 62.5% amino acid sequence identity with the SFV E1 ectodomain, the crystal structure of which is known ([18], PDB 2Ala). Unexpectedly, the far-UV spectra of the three proteins exhibited considerable differences (Fig. 4A), the spectrum of HCV E2e being in agreement with a previous study [38]. However, deconvolution to retrieve the percentage of the various secondary-structure elements suggests similar ratios for all three proteins, indicating, in particular, only about 5% α-helices in all three proteins. The strong minimum observed at 203 nm in the spectrum of HCV E2e suggests the presence of natively unfolded regions that are absent in the control proteins.

Given that circular dichroism is not the most sensitive method to determine the amount of β-sheet in a protein, we used FTIR spectrometry in a comparative analysis of E2e with a class II protein of known 3D structure. Because the WNV sE was not available at the time of the experiment, we used instead sE from dengue virus serotype 3 (DV3), for which the crystal structure is also known (PDB entry 1UZG [39]). The high-frequency region of the FTIR spectra of HCV E2e and DV3 sE is displayed in Fig. 4B. As expected, both proteins have their absorption maxima in the amide I band at 1637 and 1640 cm^−1^, respectively, close to the 1630 cm^−1^ value typical for β-sheet containing polypeptides (reviewed in [40]), in agreement with the structure of the flavivirus sE and strongly indicating that HCV E2e also contains predominantly β-sheets. In order to obtain a quantitative measure of the β-sheet content of E2e, we performed a further analysis to more precisely compare the secondary structure content of the two proteins by computing a difference spectrum after normalization to an identical area under the amide I band. The DV3/sE – HCV/E2e difference spectrum showed a positive peak at 1630 cm^−1^, as well as a broad negative region ranging from 1645 to 1680 cm^−1^ (Fig. 4B). The value of the positive peak indicated about 14% higher β-sheet content for DV3 sE, which, when using the value of 42% β-sheet estimated from the DV3 sE crystal structure, gives about 28% β-sheet for HCV E2e. The negative area of the difference FTIR spectrum indicates that the HCV E2e polypeptide displays higher relative amounts of secondary structure other than β-pleated sheet (random coil, β-turns, 3/10 helices, etc.). This difference is likely to reflect the presence of the regions that give rise to the strong minimum at 203 nm in the CD spectrum (Fig. 4A), i.e., natively unfolded segments of the polypeptide chain.

### Disulfide-bond connectivity

**Figure 5:**
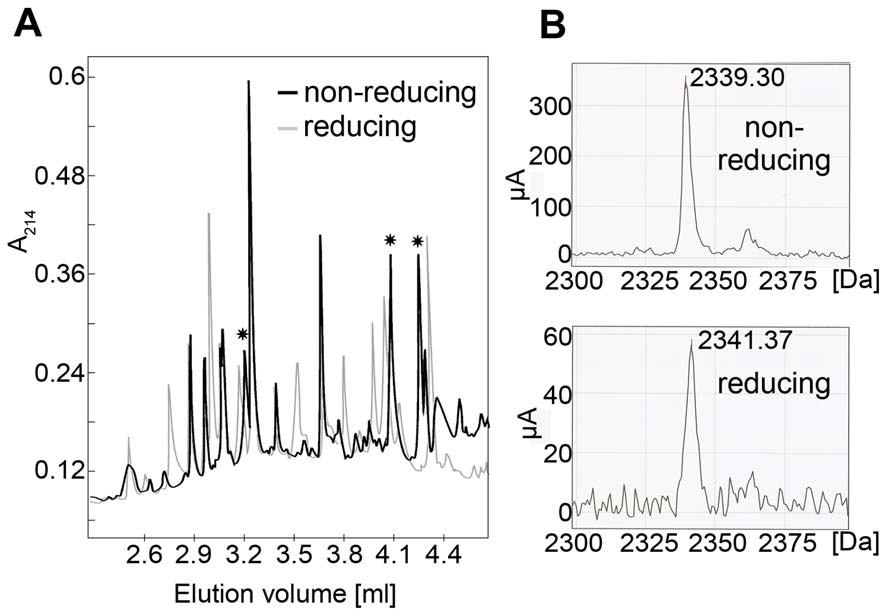
Disulfide mapping strategy. A) Typical HPLC elution profile of a tryptic digest of E2e under non-reducing (black) and reducing (light grey) conditions, superposed to highlight the difference in mobility upon reduction. Asterisks mark peaks that disappeared upon reduction and were thus selected for further proteomic analysis. B) Mass spectrum of a sample recovered from an HPLC peak susceptible to reduction (Peak 16-3 of JFH-1 E2 identified as peptide J4 in the detailed description provided in SI). Upon reduction, a shift in molecular mass of 2Da was observed, due to addition of two hydrogen atoms upon reduction of the two cysteines. The data presented in this figure allowed the identification of disulfide 4 (Table 2).

We determined the identity of the disulfide bridges by N-terminal sequencing together with comparative reducing/non-reducing mass spectrometry analyses of peptides obtained by trypsin digestion of E2e. For this purpose we selected E2e of three isolates, UKN2b_2.8, H77 and JFH-1 (genotypes, 2b, 1a and 2a, respectively), which display amino acid sequences with a different pattern of predicted trypsin cleavage sites (Fig. S2). We fully deglycosylated the protein with PNGase F under denaturing conditions, then digested it with trypsin followed by separation of the resulting peptides by HPLC under reducing or non-reducing conditions. Comparison of the HPLC elution profiles enabled the identification of peaks that were affected by reduction with TCEP (asterisks in Fig. 5A). We analyzed the samples in these peaks by surface-enhanced laser desorption/ionization (SELDI) with a time-of-flight (TOF) spectrometer (Table S1), and identified their N-terminal sequence by Edman degradation (Fig. S3). This procedure allowed the unambiguous experimental identification of 8 out of the 9 disulfide bonds present in the protein, thereby also identifying the 9^th^ by exclusion (Table 2). This table also shows that 5 disulfides were independently identified in at least two different strains, validating the procedure. A full account of the experiments made to determine the disulfide connectivity is provided as Supplementary Information (Text S1).

**Table 2:** Recapitulation of the 9 disulfide bridges and the isolates in which they were identified.

	H77	JFH-1	UKN2b_2.8
1 Cys429 - Cys552			**+**
2 Cys452 - Cys459	**+**	**+**	
3 Cys486 - Cys494	**+**		**+**
4 Cys503 - Cys508		**+**	**+**
5 Cys564 - Cys569	**+**		
6 Cys581 - Cys585	**+**		**+**
7 (Cys597 - Cys620)			
8 Cys607 - Cys644	**+**	**+**	**+**
9 Cys652 - Cys677	**+**		

### Implications for the tertiary structure of HCV E2e

The connectivity of the disulfide bonds provides key information on distant segments of the E2 polypeptide chain that come near each other in the folded protein. This knowledge can be used in conjunction with other available data to get a better picture of the tertiary structure of the protein, namely: i) the observation that E2e is rich in β-sheet and that secondary structure predictions suggest regions with consensus β-strands along its amino acid sequence; ii) the identity of residues that are far apart in primary structure and that are known to be part of the CD81 binding site; iii) the postulate that E2 is the HCV fusion protein and therefore has a characteristic 3-domain class II fold, in agreement with the organization of its precursor polyprotein, which also implies iv) that the third domain (DIII) should be connected to DI via a linker that can extend to stabilize a post-fusion trimer. Finally, DIII should be followed by a flexible “stem” region - the presence of which has already been reported for HCV E2 [41] - connecting to the TM segment. Further information comes from the identification of “hypervariable” regions in HCV E2 that can be deleted without affecting the reactivity of the resulting deletion mutant with conformation-sensitive mAbs and with CD81 [14]. In addition, numerous reports have shown that the E2 ectodomain truncated at position 661, which is in the loop closed by disulfide 9 (Table 2), also reacts with conformation-sensitive mAbs and CD81 [38], suggesting that the downstream segment is not part of the structured ectodomain.

About one third of the E2e residues are predicted to form β-strands (Fig. 3), which is in overall agreement with the estimated 28% β-sheet content determined by FTIR spectroscopy. The pattern of predicted β-strands offers the possibility of threading the polypeptide chain along the template provided by the known fold of class II proteins, while simultaneously respecting all of the known constraints derived for HCV E2 by the functional studies discussed above. A useful guide for this analysis is the comparison between predicted and experimentally observed β-strands in the crystal structure of alpha- and flavivirus fusion proteins - for instance, in the alphavirus E1 alignment provided in Fig. 3B.

**Figure 6:**
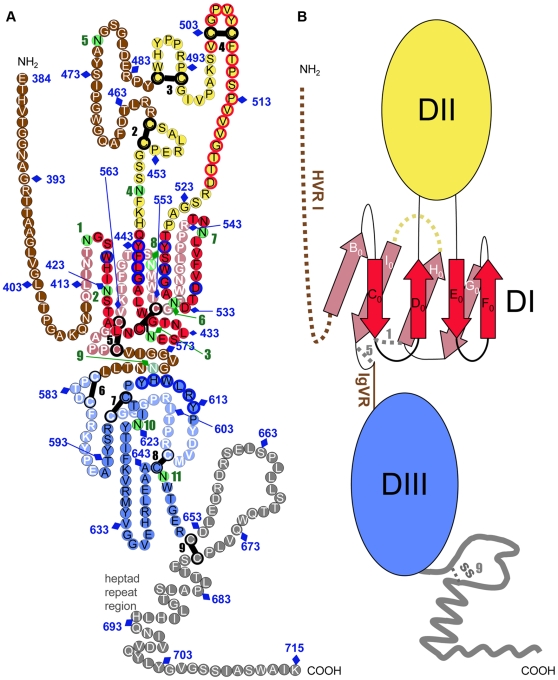
Tertiary organization of HCV E2e. A) The linear sequence of HCV H77-E2e, numbered every 10 residue according to the polyprotein (N-terminus at position 384 and the TM region beginning at 716) is represented as a chain of beads (colored circles) labeled with the corresponding amino acid and threaded onto a class II fold as described in the text. Circles in pale and bright colors represent residues in the background and foreground of the domains, respectively labeled in white and black fonts. Disulfide bonds and glycosylation sites are indicated by thick black bars and green circles, respectively, numbered sequentially. Unstructured segments are in white font on a brown background. Residues that participate in CD81 binding are contoured in blue, and those from the putative fusion loop region in red. In DIII, a plausible arrangement of the 3 predicted β-strands is illustrated by placing the corresponding polypeptide segments in an antiparallel putative β-sheet, but unlike DI, the topological arrangement of this domain was not determined. B) Schematic diagram of the tertiary organization of HCV E2, with DI, DII and DIII in red, yellow and blue, respectively. The stem region is grey. The connectivity of the β-strands in DI is indicated, labeled with the standard class II nomenclature. A broken yellow line indicates the place of the second insertion in other class II proteins.

The hallmark of the tertiary structure of class II proteins is the presence of an 8-stranded (B_0_ through I_0_) central domain (or DI) folded as a β-sandwich with up-and-down topology (Fig. 6). Two insertions in this domain, in the D_0_E_0_ and H_0_I_0_ loops, constitute the fusion domain bearing the fusion loop in the distal part of the D_0_E_0_ insertion. DI is followed, after strand I_0_, by a flexible segment connecting to a third domain (DIII), the relocation of which is important for hairpin formation during the fusogenic conformational rearrangement of class II fusion proteins.

### Threading the HCV E2 polypeptide chain on the tertiary structure of a class II template

Functional studies have shown that deletion of the HVR1 region did not induce a loss of virus infectivity in experimentally infected chimpanzees [42], indicating that this segment cannot be part of a folded domain. We therefore began the threading process by assigning the 3 consecutive β-strands predicted immediately downstream of the HVR1 (Fig. 3, first three red boxes) to the three conserved strands in the N-terminal part of DI, i.e. B_0_, C_0_ and D_0_. These β-strands are followed by a long intervening region that is compatible with the D_0_E_0_ insertion of the class II fold. For the assignment of strands E_0_ and F_0_, the available data on the residues involved in CD81 binding (small blue circles in Fig. 3) provide valuable information, since strand E_0_ must interact with D_0_ (see diagram in Fig. 6B). Thus, assigning E_0_ and F_0_ to the two consecutive strands predicted after residue 525 brings together a patch of residues that are apart in primary structure to the same face of DI, forming the site of interaction with CD81 (Fig. 6). For the assignment of the remaining β-strands, there is crucial information provided by disulfide 1. This disulfide bond connects Cys429, at the end of strand C_0_, with Cys552 further downstream, which therefore must be at the same end of the DI β-sandwich. This means that Cys552 must be located either at the G_0_H_0_ loop, or at the end of the I_0_ strand, if the molecule is to have a class II fold (Fig. 6B). However, after strand F_0,_ there is a long strand predicted to span residues 549–555 (Fig. 3), which would have Cys552 in the middle. But the comparison of predicted versus experimentally determined β-strands of alphavirus E1 shows that, for several alphaviruses, the region of G_0_ and H_0_ is also predicted as a single long strand (Fig. 3B). Indeed, in both alphaviruses and HCV, there is a glycine residue (Gly 551 in E2e) forming a tight turn that reverses the chain orientation, going from G_0_ into H_0_ (some alphaviruses have two glycines at this β-turn). This shows that the prediction algorithms are not 100% reliable, suggesting that in HCV E2, Gly551 is at the G_0_H_0_ turn, and that Cys552 is the first residue of strand H_0_. Indeed, running at the edge of the DI β-sandwich, the sequence of G_0_ (as well as the sequence of the alphavirus B_0_ strand, at the other end of the bottom β-sheet, Fig. 3B) appears to be less typical than the sequences of internal β-strands in a β-sheet, which are easier to predict by computer algorithms. In addition, the short connections between strands F_0_ through H_0_ in both alpha- and flavivirus DI are also consistent with the assignment of H_0_ to a strand running between residues 552 and 555 in HCV E2.

Having assigned the G_0_ and H_0_ strands, additional considerations are necessary to assign strand I_0_. In alpha- and flaviviruses, I_0_ is one of the two central β-strands of the bottom sheet of DI, and is directly followed by the linker connecting to DIII. Because it is the only strand missing to complete the 8-stranded β-sandwich, it can only make disulfide bonds to cysteines located upstream in primary sequence. In HCV, three β-strands are predicted directly downstream to the assigned H_0_ strand: one around residue 563, one around 573, and one around 593 (Fig. 3). The strand around residue 573 is part of a segment that can be deleted without affecting protein conformation [14], indicating that it cannot be I_0_. The strand around 593 ends at Cys597, which forms disulfide 7 with Cys620 further downstream. Because class II proteins can have no interdomain disulfides - which would be incompatible with their function - this strand cannot be assigned to I_0_ either. Indeed, the interleaved nature of disulfide bonds 7 and 8 dictates that none of the strands predicted downstream can be in DI. The only option compatible with a class II fold is, therefore, to assign the strand around residue 563 to I_0_. This assignment implies that the long insertion in the H_0_I_0_ loop of the alpha- and flavivirus fusion proteins is absent in HCV E2. This is in line with E2 from HCV and pestiviruses being shorter than the alpha- and flavivirus fusion proteins by about 80–110 amino acids, i.e., roughly the length of the insertion in the H_0_I_0_ loop of the latter.

The assignment of the 8 strands in HCV DI also indicates that the linker connecting DI and DIII must be between disulfides 5 and 6, encompassing the region called IgVR (“intergenotypic variable region”), which can be deleted without affecting protein conformation, at least in the prefusion form of E2. As discussed above, the segment containing disulfide 9 is likely not to be part of the structured ectodomain, further implying that DIII is comprised between disulfides 6 and 9, spanning about 70 amino acids. The presence of two long-range disulfide bonds (disulfides 7 and 8) suggests that this region is indeed structured into a separate domain. However, the secondary structure predictions point to only 3 β-strands in this domain, indicating that the Ig-like fold of DIII in alpha- and flaviviruses may not have been maintained in HCV. Moreover, we found no obvious way to propose an Ig-like arrangement of the polypeptide chain in this domain such that it would also satisfy the constraints imposed by disulfides 7 and 8.

### Main features of the teriary structure of HCV E2

The resulting model for the tertiary structure of E2 is presented in Fig. 6A, with the diagram of Fig. 6B higlighting, as a guide, the essential features of the resulting “class II” organization of the protein. The main features of the molecule are the following:

### DI

This domain has an N-terminal extension in flaviviruses (which includes β-strand A_0_) with respect to alphaviruses (see review by [16]), and in HCV, the HVR1 also appears to be an N-terminal extension. DI contains disulfides 1 and 5, both at the DII distal end of the DI β-sandwich; i.e., at its DIII interacting end. Disulfide 5 connects two consecutive cysteines into a short loop at the end of strand I_0_.

The C_0_D_0_E_0_F_0_ β-sheet (or “top” sheet) contains most of the determinants of CD81 binding (blue circles in Fig. 6A), and 5 of the 11 N-linked glycosylation sites of E2 (numbered 1, 2, 3, 6 and 7, Figs. 3 and 6A). In contrast, the B_0_I_0_H_0_G_0_ β-sheet (or “bottom” sheet) has only site 8 (Asn 556, Fig. 6A), located in the H_0_I_0_ loop, at the site of the long insertion in alpha- and flavivirus fusion proteins (yellow dotted line, Fig. 6B). The presence of an insertion in the other class II proteins suggests that there is space at this end of the barrel for a glycan chain attached to Asn556. Importantly, glycan 8 was shown to be essential for the correct folding of E2, in line with the key location in the H_0_I_0_ loop in the bottom sheet. Overall, the distribution of glycans on HCV DI is compatible with the experimentally determined orientation of flavivirus E and alphavirus E1 at the virion surface, with the bottom sheet facing the viral membrane. This pattern provides additional evidence validating our assignment of the DI β-strands.

### DII

This domain has two predicted glycosylation sites and three disulfide bonds (2, 3 and 4), all connecting consecutive cysteine residues very close in primary structure. In the alpha- and flavivirus counterparts, the two insertions forming DII are quite intertwined, and the second one (the H_0_I_0_ insertion) acts as a scaffold supporting the D_0_E_0_ insertion bearing the fusion loop at the DI-distal end. In HCV E2, the absence of the second insertion makes DII much smaller, and apparently also results in a more flexible, or disordered domain, as suggested by the absence of long-range disulfide bonds and the fact that the whole area between disulfides 2 and 3 can be eliminated without affecting the overall conformation of the molecule.

### A putative fusion loop

The proposed tertiary organization of E2 provides a prediction for the location of the HCV E2 fusion loop, which in class II fusion proteins is a stretch of highly conserved residues within the D_0_E_0_ insertion. This segment is composed mainly of non-charged residues, and is rich in glycine and non-polar amino acids. The sequence alignment highlights the region spanning residues 502–520 (red circles in Fig. 6A), which has similar characteristics and is strictly conserved within all HCV genotypes (Fig. 3). This region is thus a strong candidate for fulfilling the role of the HCV fusion loop. The smaller conserved block between residues 484–489 has been tested by site directed mutagenesis using retroviral particles pseudotyped with the HCV envelope proteins (HCVpp), suggesting that it does not play a role in membrane fusion [29]. Because in all class II fusion proteins, the fusion loop is buried at an oligomeric interface in the prefusion form, the candidate fusion loop segment is also very likely to mark a contact region with E1.

### Linker region and DIII

Our model predicts that DIII (blue), which in alpha- and flavivirus fusion proteins has an Ig-like fold, contains disulfides 6, 7 and 8, and the last two glycosylation sites, 10 and 11. It is connected to the C-terminal end of DI via the IgVR, which contains glycan 9. This linker region is such that it can be extended to allow the translocation of DIII to the side of the trimer during the fusogenic conformational change, as expected for a class II fusion protein. Yet this segment, located between disulfides 5 and 6 (highlighted in brown in Fig. 6A), can also be replaced by a GSSG linker without affecting the overall protein conformation [14], suggesting close apposition between DI and DIII at least in the prefusion form. This organization is also compatible with the observation that some of the residues important for CD81 binding map to DIII (613–618, Figs. 3 and 6), suggesting that CD81 bridges the surface of the two domains. Indeed, these two domains display an extended interaction surface in other class II fusion proteins.

### The stem region

DIII is followed in sequence by a relatively flexible but conserved region, denoted the “stem” (grey in Fig. 6), which connects to the TM segment. The stem would contain a loop that is closed by the last disulfide (number 9). A number of reports on the characterization of a protein ending at position 661 indicate that the absence of this loop does not affect the conformation of the protein and it is therefore not part of DIII. Further support for this interpretation is provided by our identification of a trypsin-resistant fragment of E2e ending at position Arg648, in between disulfides 8 and 9 (data not shown).

### Implications of the tertiary structure model of HCV E2

One important implication is that the residues interacting with CD81 are found in two domains, DI and DIII, which have to move apart during the fusogenic conformational change. This suggest that CD81 may have to dissociate away for such a conformational change to take place, or on the contrary, that its binding may help to lower the energy barrier for the conformational change to occur upon exposure to low pH in the endosomes.

An additional information from this study is the positioning of the hypervariable regions of HCV E2 in the context of the class II fold. As suggested above, the presence of these regions is likely to be responsible for the difference in CD spectrum between HCV E2e and the other class II proteins examined (Fig. 4A), which do not contain unstructured regions. Such regions are presumably important for evading the humoral immune response of the host, given that HCV can cause chronic infection, in contrast to alpha- and flaviviruses. Another important difference to the latter are the numerous glycosylation sites in HCV E2. Our model indicates that these sites cluster in particular on the exposed face of DI. Indeed, several glycans appear to frame the CD81 binding surface, partially shielding it from recognition by circulating antibodies. These are glycans 1, 2, 6, 7, and possibly 10 and 11 (Fig. 6A). Importantly, a number of studies have pointed to a role of some of these E2 glycosylation sites modulating entry and/or CD81 binding [43],[44].

Another important implication is the identification of a strong candidate region for the fusion loop. This polypeptide segment, spanning residues 502–520 of E2, has all the characteristics reported for the experimentally characterized class II fusion loops. It is strictly conserved, and is located in a region of the protein that is compatible with this function – in the D_0_E_0_ insertion of DI - in spite of being part of a fusion domain (DII) that is much smaller than its alpha - and flavivirus counterpart. The fact that in the latter DII is formed by two insertions into a simple, conserved DI, suggests that they may have evolved sequentially. HCV may have thus maintained some ancient intermediate form of the class II proteins, containing only the insertion that carries the fusion loop.

The observed flexible and largely unstructured conformation of DII is likely to be due to the absence of the H_0_I_0_ scaffold. The presence of the candidate HCV fusion loop relatively close to DI is another important difference. In the alpha- and flavivirus counterparts, there are about 30 intervening residues present in an extended conformation between the fusion loop and strand E_0_, whereas there are only a few residues in HCV. This difference is likely to be related to the intrinsic flexibility of HCV DII, since the fusion loop is unlikely to lie at the distant tip of an unstructured domain. During membrane fusion, the fusion loop could be further stabilized by interaction with the membrane proximal region of the stem, once the molecule adopts its fusogenic hairpin conformation. This organization also suggests a significantly shorter post-fusion HCV E2 trimer, compared to the other class II proteins. This would be analogous to the observed differences in the post-fusion trimers of class I viral fusion proteins, for instance from retroviruses, which are short [45], and paramyxoviruses [46] or coronaviruses [47], which display a very long hairpin conformation.

Overall, our model for the HCV E2 tertiary organization provides a structural framework to understand the antigenicity of the virion, the organization of the regions that interact with CD81, and the putative conformational changes that are likely to take place during the membrane fusion reaction to invade a target cell. In the absence of 3D structural data, our results constitute an important step to better understand the function of the HCV envelope proteins. This knowledge, in turn, can help devise possible antiviral strategies against this important pathogen. Our data also provide a handle to dissect and obtain structural data on the E2 domains separately, given that the intact ectodomain is very difficult to crystallize.

Finally, this analysis highlights the power of conducting parallel structural studies on related viruses, which provide information that can be extrapolated to other members of the respective viral families, even in the absence of sequence similarity in the corresponding proteins.

## Materials and Methods

The accompanying Supplementary Information (Text S1) describes in detail the construction of the vectors used for expression of synthetic genes coding for the E1-E2ΔTM segment of the 9 HCV isolates tested (Table 1), the protocols for production, purification and conformational characterization of recombinant HCV E2e, as well as the computer and experimental analyses used for secondary structure predictions. Finally, a detailed description of the procedures used for the experimental identification of the cysteine residues involved in 8 disulfide bonds of E2 is provided.

## Supporting Information

Text S1 Supplementary materials and methods
(0.15 MB PDF)

Table S1 Identification of disulfide bond connected HCV E2e peptides resulting from tryptic digest
(0.05 MB PDF)

Figure S1 Conformational characterization of HCV E2e. A) Pull-down experiment showing that E2e specifically reacts with conformation dependent antibodies CBH-4B and CBH-4D, but not a control antibody, and binds the CD81 LEL. E2e was affinity loaded onto a Streptactin column, samples containing the respective proteins were passed through the column and the complex was eluted after washing. Elution fractions were analysed by SDS-PAGE under reducing conditions. Bands representing the E2e, CD81-LEL as well as the heavy chain (HC) and light chain (LC) of the two antibodies were observed. B) Stoichiometric complex formation between UKN2b_2.8 E2e and mAb CBH4D. UKN2b_2.8 E2e, mAb CBH4D and a mixture of the two (ratio 2:1) were loaded to the column (in three different runs) (E2e∼50kD, H53∼150kD, complex∼250kD). No peaks corresponding to either of the isolated proteins were observed in the profile of the complex, indicating a 2:1 complex stoichiometry and a high affinity of UKN2b_2.8 E2e for mAb CBH4D.
(1.23 MB TIF)

Figure S2 Alignment of HCV E2 amino acid sequences from strains H77, JFH-1 and UKN2b_2.8. Given the deamination of Asn residues by PNGase F, they are turned into Asp residues. Predicted trypsin cleavage sites (grey triangles) and N-glycosylation sites (empty diamonds) are indicated, cysteines are boxed and the respective disulfide bridges displayed (-SS-). Peptides identified after tryptic cleavage are shaded, named according to the respective isolate and numbered sequentially following the amino acid sequence of E2.
(2.52 MB TIF)

Figure S3 Proteomics results for the determination of the disulfide bridges. HPLC chromatogram peaks that were selected for further proteomics analysis. Results of N-terminal sequencing and SELDI-TOF MS for the respective peaks are shown in panels A-G. Scales for the intensity (y-axis) as well as for the molecular weight (x-axis) vary considerably in the different spectra, resulting in a different appearance of the background noise. A) Peaks JFH-1 6-3 and 12-3, leading to the identification of disulfide bridges 2 (peak 6-3) and 8 (peak 12-3) in E2e of JFH-1. B) Peak JFH-1 16-3, leading to the identification of disulfide bridge 4 in E2e of JFH-1. C) Peaks UKN2b_2.8 13-1 and 20-1, leading to the identification of disulfide bridges 2 (peak 20-1) and 8 (peak 13-1) in E2e of UKN2b_2.8. D) Peaks UKN2b_2.8 42-3 and 19-1, leading to the identification of disulfide bridges 1 (peak 42-3) and 6 (peak 19-1) in E2e of UKN2b_2.8. E) Peaks H77 15-2 and 6-2, leading to the identification of disulfide bridges 2 (peak 6-2) and 8 (peak 15-2) in E2e of H77. F) Peaks H77 26-2, which is the result of disulfide shuffling, and 32-2, leading to the identification of disulfide bridge 3 in E2e of H77. G) Peaks H77 43-2 and 33-2, leading to the identification of disulfide bridges 5 (peak 33-2) and 9 (peak 43-2) in E2e of H77.
(6.93 MB TIF)
