# Supplementary material for: The Disulfide Bonds in Glycoprotein E2 of Hepatitis C Virus Reveal the Tertiary Organization of the Molecule
Source: PLoS Pathog. 2010 Feb 19;6(2):e1000762. doi: 10.1371/journal.ppat.1000762 (PMC2824758; doi:10.1371/journal.ppat.1000762)
Supplement: Text S1 — Supplementary materials and methods (0.15 MB PDF) [file ppat.1000762.s001.pdf]

SUPPLEMENTARY INFORMATION TO MANUSCRIPT:

The Disulfide Bonds in Glycoprotein E2 of Hepatitis C Virus Reveal the Tertiary Organization of the Molecule

Thomas Krey[1,2,4], Jacques d'Alayer[1,3], Carlos M. Kikuti[1,2,4], Aure Saulnier[1,5,4], Laurence Damier-Piolle[1,2,4], Isabelle Petitpas[6], Daniel X. Johansson[7,¶], Rajiv G. Tawar[1,2,4], Bruno Baron[1,8], Bruno Robert[9], Patrick England[1,8], Mats A. A. Persson[7], Annette Martin[1,5,4] and Félix A. Rey[1,2,4,*]

[1]Institut Pasteur, 25 rue du Dr. Roux, 75724 Paris Cedex 15, France, [2]Unité de Virologie Structurale [3]Plate-forme d'Analyse et de Microséquençage des Protéines, [4]Départment de Virologie, CNRS URA 3015, [5]Unité de Génétique Moléculaire des Virus à ARN, [6]Laboratoire de Virologie Moleculaire et Structurale, UMR CNRS 2472/INRA 1157, 91198 Gif-sur-Yvette, France, [7]Department of Medicine, Center for Molecular Medicine, Karolinska University Hospital, 171 76 Stockholm, Sweden, [8]Plate-forme de Biophysique des Macromolécules et de leurs Interactions, CNRS, URA 2185, [9]Institut de Biologie et de Technologie de Saclay, CEA, and URA 2096, CNRS, 91191 Gif-sur-Yvette, France

[¶]Present address: Department of Microbiology, Tumor and Cell Biology, Karolinska Institutet S-171 77 Stockholm, Sweden

This document includes: **Page**

Materials and Methods	2
Supplemental References	14

Materials and Methods

Cells, viruses and media

Drosophila Schneider 2 cells were purchased from Invitrogen and cultured at 28°C in Schneider's Drosophila medium (Invitrogen, Carlsbad, USA). Stable cell lines were transferred to serum-free Insect Xpress media (Lonza, Basel, Switzerland), which was also used for protein production. Human hepatoma cells (Huh 7.5) [1] were cultured in Dulbecco's Modified Eagle Medium supplemented with 10% fetal bovine serum (FBS), 100 U/ml penicillin, 100 µg/ml streptomycin, and a 100µM mixture of non-essential amino acids (DMEM-10%, all reagents from Invitrogen, Cergy-Pontoise, France) and maintained at 37°C in a 5% CO_2 atmosphere.

HCV stocks (HCVcc) were produced after electroporation of cB76.1/Huh7 cells [2] with RNA transcripts synthesized *in vitro* from pJFH1 [3]. Cell supernatants were harvested at 7 days post-transfection and virus titers (in focus forming units (FFU)/mL) were determined by indirect immunofluorescence on Huh7.5 cells, as described below.

Plasmids

We modified the pMT/BiP vector (Invitrogen) to encode for an engineered E2e protein such that it can be efficiently purified upon induction of the metallothionein promoter with divalent cations (Cu^{2+} or Cd^{2+}). The original vector encodes the *Drosophila* BiP signal sequence (SS) at the N-terminus of the construct for efficient translocation into the ER of the S2 cells, in frame with the gene of the secreted protein of interest (replacing the endogenous SS). In order to avoid interference of

the divalent metal ions used for induction with the histidine tag used for purification of the secreted protein, we replaced the region including the V5 epitope and the 6-Histidine tag by a segment coding for a specific proteolytic cleavage site, followed by a tandem strep-tag (IBA, www.iba-go.com) with a linker region $(GlyGlySer)_4$ in between. The proteolytic cleavage site was added to allow the specific removal of the tag for structural studies. We avoided the use of cysteine proteases (like Prescission, TEV or 3C proteases) which, although are very specific, require a reducing agent for activity, which could also reduce some of the exposed disulfides of the glycoprotein. We instead engineered an enterokinase (EK) cleavage site, which is a serine-protease relatively specific for the sequence $(Asp)_4Lys\downarrow X$, cleaving at the site indicated by the arrow with a cleavage efficiency between 60 and 80 % (X being any amino acid) [4]. This resulted in the following amino acid sequence downstream of the ApaI and BstBI sites ...DDDDKAGWSHPQFEKGGGSGGGSGGGSWSHPQFEK-COOH. All synthetic HCV glycoprotein genes were purchased from GeneCust (Dudelange, Luxemburg) and amplified by PCR using strain specific 5'-oligonucleotides containing Bgl II, which allows insertion immediately downstream of the BiP secretion signal, and strain specific 3'-oligonucleotides containing Apa I.

A full list of oligonucleotides used in this study is available upon request

Generation of inducible Drosophila S2 cell lines producing E2e

Transfection of *Drosophila* S2 cells was done using Effectene (Qiagen, Hilden, Germany) according to the manufacturers recommendations, with 2µg of the respective plasmids. A second plasmid, encoding either Blasticidin S deaminase or

puromycin acetyltransferase, respectively, was cotransfected as dominant selectable marker. Stable E2e expressing cell lines were selected by addition of 6µg/ml Puromycin or 25µg/ml Blasticidin S (Invivogen, San Diego, USA) to the culture medium 72h after transfection. Adaptation of the cell lines to serum free Insect Xpress media was performed stepwise as recommended by Invitrogen. Expression systems for West-Nile virus E protein (aa 1-442 of the E protein), Dengue virus serotype 3 E protein (aa 1-392), E2 of bovine viral diarrhea virus (aa 1-339) as well as for E1 of Chikungunya virus (aa 1-381) were designed in a similar way.

Production and purification of envelope glycoprotein

For large scale production of E2e the cells were cultured in spinner flasks or in Wave Bioreactors (2/10, Wave Biotech, Somerset, USA) and induced with 4µM $CdCl_2$ at a density of approximately $7x10^6$ cells per ml. After 8 days at 28°C cells were pelleted and E2e was purified by affinity chromatography from the supernatant using a StrepTactin Superflow column (IBA, Goettingen, Germany) followed by gel filtration chromatography using a Superdex200 column (GE Healthcare, Uppsala, Sweden). Pure protein was quantified using adsorption at UV_{280nm} and concentrated to approximately 1 mg/ml.

SEC analysis of E2e – antibody complexes

25µg of H77 E2e and 70µg of either mAb H53 or CBH-4D, respectively, were incubated as isolated proteins as well as in complex for 1h at 10°C followed by analysis on a Superdex200 Mini column (column volume 3 ml, Amersham).

Pull-down assay of antibodies and CD81 using E2e

25μg of E2e was bound to a StrepTactin Superflow mini column (column volume 0.2ml) and washed with 10 column volumes of washing buffer. Subsequently, 10μg of CD81 large extracellular loop (produced as described before [5]) or 50μg of conformation dependent antibodies CBH-4B, CBH-4D against HCV E2 (kindly provided by S. Foung, Stanford, USA) or a control antibody were added, followed by washing with 10 column volumes. Complexes were eluted in 4.5 column volumes elution buffer and concentrated 20-fold by ultrafiltration. This concentrate was analysed by SDS-PAGE and Coomassie Blue staining.

Inhibition of HCVcc infection by E2e

Huh7.5 cells plated on glass coverslips in 24-well plates (4.5×10^4 cells per well) were incubated for 1h at RT in the absence or presence of increasing concentrations (0.05-2μM) of HCV E2e, BVDV E2e or West Nile virus sE used as controls. Cells were subsequently washed and infected with 7×10^3 FFU of HCVcc in the presence of identical concentrations of the respective viral glycoproteins. After a 5h adsorption period at 37°C, the viral inoculum was removed and replaced with fresh medium. At 3 days post-infection, cells were fixed with 4% paraformaldehyde, permeabilized in 0.2% Triton X-100 in PBS, and subsequently processed for core detection by indirect immunofluorescence using 0.5 μg/mL of anti-core monoclonal antibody 1851 (Abcys, Paris, France) and Alexafluor 555-conjugated anti-mouse IgG (Invitrogen) at a 1:500 dilution. Coverslips were mounted on slides using ProLong Gold Antifade Reagent with DAPI (Invitrogen) allowing counterstaining of

cell nuclei. Virus titers were determined by counting labeled foci following acquisition of mosaic images spanning the entire surface of each coverslip using an Axioplan2i microscope (Zeiss) with a Wide Field ApoTome (Zeiss) and the Axiovision software (Zeiss). The mean value of all three concentrations (0.5µM, 1µM and 2µM) of both control proteins was taken as 100%.

Far-UV circular dichroism of envelope glycoproteins

Soluble HCV E2, Chikungunya virus E1 and West-Nile virus E protein at a concentration of 0.7mg/ml in 20mM phosphate pH 7.5, 150mM NaF were analysed by Far-UV circular dichroism (180-260 nm). CD spectra were obtained with an AVIV CD spectropolarimeter model 215 using a 0.02cm path length cell at room temperature. Five successive scans were averaged and the background spectrum of the sample buffer, acquired under identical conditions, was subtracted. The resulting corrected CD intensities were then converted to $\Delta\varepsilon$ per residue. Secondary structure contents were estimated from the far-UV CD spectra using the CDSSTR routine [6] of the DICHROWEB server [7,8] run on the SP175 reference dataset [9], containing 72 proteins representing a large panel of secondary structures. Similar results were obtained on different datasets [10] or by using the CONTIN/LL routine [11].

Fourier transform infrared spectroscopy of envelope glycoproteins

Soluble HCV E2 and Dengue virus 3 E protein in a concentration of 5-10mg/ml in 10mM Tris pH 8.0, 150mM NaCl were used for FTIR analysis. Attenuated total

reflectance FTIR spectra were measured with a Bruker vector 22 spectrophotometer equipped with a 45° diamond ATR attachment (National Instruments, U.K.). The spectra shown represent the average of 300 scans after removal of the buffer signal. In order to obtain a difference spectrum the spectra were normalized with respect to the total area under the amide I band and the difference was calculated by subtraction of the HCV E2 spectrum from the spectrum of DV3 sE.

Deglycosylation and proteolytic digestion of E2e

200μg of E2e of the three isolates H77, JFH-1 and UKN2b_2.8 (genbank accession numbers GI:130461, GI:116078059 and AY734983, respectively) were boiled 10 minutes at 95°C in 2.5% SDS and the denatured protein was incubated with His-tagged PNGase F in excess at 37°C overnight. Subsequently, PNGase F was removed by ion metal affinity purification. The deglycosylated protein was concentrated to approximately 1.5mg/ml and analysed by SDS-PAGE followed by staining with Amido Black. Bands containing approximately 15µg E2e were cut out of the gel and subsequently digested at 37°C in 0.01% Tween20, 50mM TrisHCl with 0.5µg Trypsin. In order to stabilize existing disulfide bonds the pH of the reaction was set to the lowest possible value (pH 7.0 for experiment 1, pH 7.6 for experiment 2 and pH 8.6 for experiment 3), which resulted in efficient digestion. After 8h, 0.25µg of trypsin were added and the digestion was continued for another 16h. The peptides were eluted from the gel using 200µl water and two times 100µl 60% Acetonitril.

As a control, the trypsin digestion experiment was carried out as described above, but in the presence of 5% DMSO, acting as oxidizing agent. The deglycosylated

protein was analysed by SDS-PAGE followed by transfer onto a Nitrocellulose membrane and staining with Poinceau-Red. Bands containing approximately 15µg E2e were cut out of the membrane and saturated with 1ml of 0.2% PVP K30 for 15 minutes followed by six washes, four with water and two with 50mM TrisHCl pH 7.6. Subsequently the protein was digested with 1µg Trypsin for 3h at 37°C in the absence or presence of 5mM NEM. The peptides were eluted from the membrane using 200µl of water.

HPLC of resulting peptides and subsequent proteomic analysis

The tryptic digest was divided in two half fractions. One half was submitted directly to reverse-phase HPLC using DEAE-C18 columns (1mm diameter) and a gradient of 2 to 70% Acetonitrile in TFA 0.1%. The second half was reduced by addition of TCEP to a final concentration of 2.5mM (for the control experiment including NEM 10mM TCEP were used) for 30 minutes at RT and prior to reverse-phase HPLC under identical conditions (Fig. 5A). Peptides present in fractions corresponding to peaks of the HPLC chromatogram made under non-reducing conditions were serially numbered, and peaks that disappeared upon reduction were selected for further analysis by N-terminal sequencing and Surface-Enhanced Laser Desorption/Ionisation - Time-Of-Flight Mass Spectrometry (SELDI-TOF MS) using H4-protein chips (Ciphergen, Goettingen, Germany).

N-terminal sequencing and SELDI-TOF analysis

N-terminal sequencing was performed using a ABI 494 Protein Sequencer (Applied BioSystems, Foster City, USA). SELDI-TOF analysis was performed on a Protein

ChipReader System 4000 using a H4 (reversed phase, Ciphergen Biosystems, Fremont, CA, USA) surface and a SPA matrix, which was prepared according to the manufacturer's instructions. Peak identification was carried out using ProteinChip Software 3.1 (Ciphergen). Molecular weight prediction of disulfide-connected peptides was performed using MS-BRIDGE [12], while molecular weight of reduced peptides was predicted using PeptideMass [13].

Determination of disulfide connectivity pattern in HCV E2e

In order to identify the disulfide bridges of HCV E2e we first performed a tryptic digestion of E2e of the JFH-1 isolate. The HPLC chromatogram of the resulting digest revealed peaks 6-3, 12-3 and 16-3 to be TCEP sensitive and disappear upon reduction.

Peak 6-3 revealed a mixture of peptides, the N-terminal sequencing of which showed that only J1 and J2 (Table S1 and Fig. S3A) contained a cysteine residue (position 452 and 459, respectively). In the respective mass spectrum a peak corresponding to the disulfide linked dipeptide could be identified (1471.71 Da), which disappeared upon reduction (Fig. S3A), indicating a disulfide bridge between Cys452 and Cys459.

Peak 12-3 contained peptides J6 and J7, each of them with one cysteine (position 607 and 644, respectively). While peptides J6 and J7 were found as single peptides in the mass spectrum, indicating partial reduction, we also observed a peak at the predicted molecular weight of the two peptides linked by a disulfide bond (Fig. S3A, 2045.37 Da). This peak disappeared, as expected, upon reduction. This clearly suggested a disulfide bridge between Cys607 and Cys644.

Peak 16-3 contained a mixture of peptides with one dominant sequence corresponding to peptide J4, containing two cysteines (position 503 and 508, respectively) and a proline residue in between. This peptide was unambiguously identified in the mass spectrum of peak 16-3 (Fig. 5B, Fig. S3B and Table S1, 2341.37 Da). Reduction with TCEP resulted in a molecular weight shift by 2 Da, which was interpreted as two hydrogen atoms added upon reduction of the cysteines, demonstrating an intrapeptidic disulfide bridge between Cys503 and Cys508. Two more peptides, which could not be observed by mass spectrometry, were identified by N-terminal sequencing in peak 16-3: peptide J3 and peptide J5, containing Cys486 and Cys494 as well as Cys581 and Cys585, respectively.

Subsequently we subjected the E2e from isolate UKN2b_2.8 to trypsin digestion. HPLC separation of the resulting peptides revealed that peaks 13-1, 20-1, 29-3, 42-3 and 19-1 were TCEP sensitive and disappeared upon reduction.

N-terminal sequencing identified two peptides, U6 and U7, containing Cys607 and Cys644, in peak 13-1 (which correspond to J6 and J7, see Table S1), confirming the data obtained with JFH-1 E2e. Mass spectrometry of U6 and U7 confirmed the presence of this disulfide bond in the UKN2b_2.8 isolate (Fig. S3C, 2037.59 Da). However, the observed partial reduction of this disulfide bond in both strains suggested that experimentally induced disulfide shuffling may have occurred during trypsin digestion. In order to assess this, we performed three different control experiments limiting this effect: (1) in-gel digestion in the presence of 5% DMSO, which acts as oxidizing agent, (2) digestion on a Nitrocellulose membrane in order to reduce incubation time to 3h in the absence or (3) in the presence of NEM (*N*-ethylmaleimide), which covalently binds to free cysteines and thus blocks any disulfide rearrangements. The disulfide bridge between Cys607 and Cys644 was

observed by N-terminal sequencing in all three control experiments (data not shown), strongly suggesting that it is also present in the native protein.

Peak 20-1 contained exclusively peptide U3, which corresponds to peptide J4 in JFH-1 E2e, thereby confirming the presence of a disulfide bridge between Cys503 and Cys508 (Table S1 and Fig. S3C, 2194.94 Da).

Analysis of peak 29-3 revealed two TCEP sensitive peptides, U2 and U3. We had identified U3 previously to carry an internal disulfide bridge, thus suggesting an additional internal disulfide bond between Cys486 and Cys494 in peptide U2.

Peak 42-3 contained a mixture of three different peptides: U1, U3 and U4. Previous experiments showed that the two cysteines in U3 (Cys503 and Cys508) form an intrapeptidic disulfide bridge. Since U1 and U4 each contain one cysteine (position 429 and 552, respectively) this suggested a disulfide bond between Cys429 and Cys552. Although the disulfide linked peptides could not be identified by mass spectrometry, upon reduction a peak corresponding to the reduced peptide U1 was observed (Fig. S3D, 2308 Da). Likely the high molecular weight of the disulfide linked dipeptide (U1 + U4 - 6890.68 Da) prevented its appearance in the spectrum.

One peak (19-1) was found to contain a mixture of sequences, with one dominant sequence corresponding to peptide U5, in which two cysteines (position 581 and 585) are present. We observed a peak corresponding to the peptide harboring an intrapeptidic disulfide bridge in the mass spectrum (Table S1 and Fig. S3D, 1849.64 Da). Reduction resulted as expected in an increase of the molecular weight by 2 Da. In addition, peptide U2 was found in the same peak, which has previously been shown to carry an intrapeptidic disulfide bond.

Finally, we performed a tryptic digestion of the of E2e of H77 followed by HPLC of the resulting peptides, which revealed that peaks 15-2, 6-2, 26-2, 32-2, 43-2 and 33-2 disappeared upon reduction.

Peptides H5 and H6, which correspond to J6/ J7 and U6/ U7 were identified in peak 15-2. For both E2 of JFH-1 and UKN2b_2.8 a disulfide bridge between the respective cysteines (position 607 and 644) was shown in this study. Mass spectrometry clearly demonstrated the presence of a disulfide bridge between Cys607 and Cys644 in the ectodomain of H77 E2 as well (Table S1 and Fig. S3E, 2110.35 Da).

Peptide H1 was found in two different peaks. Together with peptide H2, which corresponds to peptide J2, it was observed in peak 6-2, suggesting the presence of a disulfide bridge between Cys452 and Cys459, which has already been identified in E2e from strain JFH-1. A peak in the mass spectrum corresponding to this peptide confirmed the presence of this disulfide bond (Fig. S3E, 1544.29 Da). However, peptide H1 was also found together with peptide H6 in peak 26-2, which clearly suggested a disulfide rearrangement for these cysteines (Fig. S3F). In order to verify the actual disulfide bonding partner of Cys452 present in H1, the three control experiments mentioned above were performed. In all control experiments the disulfide bridge between peptides H1 and H2 was observed by N-terminal sequencing, while the presence of peptides H1 and H6 in the same peak disappeared under the conditions of the control experiments. Thus we conclude, that Cys452 is effectively linked to Cys459.

In peak 32-2 we found only peptide H3, which corresponds to U2 (Table 1), in which we had already identified an intrapeptidic disulfide bond. Mass spectrometry

confirmed the presence of this disulfide bond between Cys486 and Cys494 (Fig. S3F, 4321.98 Da).

Peak 43-2 consisted of the peptides H7 and H8, each containing one cysteine residue (position 652 and 677, respectively). In the mass spectrum we observed a peak corresponding to the disulfide linked dipeptide (Fig. S3G and Table S1, 6849.91 Da), unambiguously identifying a disulfide bridge between Cys652 and Cys677 in the ectodomain of H77 E2.

Comparing the sequence alignment of E2 in the region between Cys569 and Cys581 we noticed that while UKN2b_2.8 and JFH-1 E2 contain three trypsin cleavage sites, H77 E2 has no cleavage sites in this region (Fig. S2). Thus trypsin cleavage prediction in this region resulted in one peptide containing 4 cysteines, aligned sequentially in a way that the first two cysteines and the last two each have a proline residue in between. Analysis of peak 33-2 revealed only peptide H4, which corresponds to the predicted peptide containing 4 cysteines (positions 564, 569, 581 and 585, respectively). Mass spectrometry revealed a peak matching the predicted mass of this peptide containing two intrapeptidic disulfide bridges (Fig. S3G, 2504.50 Da). Under non-reducing conditions two minor peaks could be observed, which are shifted by exactly 2 Da and thus likely correspond to partially reduced peptides in the original HPLC peak. Since we had already identified the disulfide bond between Cys581 and Cys585 in UKN2b_2.8 E2, this result strongly indicates the presence of a disulfide bridge between Cys564 and Cys569.

References:

1. Blight KJ, McKeating JA, Rice CM (2002) Highly permissive cell lines for subgenomic and genomic hepatitis C virus RNA replication. J Virol 76: 13001-13014.

2. De Tomassi A, Pizzuti M, Graziani R, Sbardellati A, Altamura S, et al. (2002) Cell clones selected from the Huh7 human hepatoma cell line support efficient replication of a subgenomic GB virus B replicon. J Virol 76: 7736-7746.

3. Wakita T, Pietschmann T, Kato T, Date T, Miyamoto M, et al. (2005) Production of infectious hepatitis C virus in tissue culture from a cloned viral genome. Nat Med 11: 791-796.

4. Hosfield T, Lu Q (1999) Influence of the amino acid residue downstream of (Asp)4Lys on enterokinase cleavage of a fusion protein. Anal Biochem 269: 10-16.

5. Kitadokoro K, Bordo D, Galli G, Petracca R, Falugi F, et al. (2001) CD81 extracellular domain 3D structure: insight into the tetraspanin superfamily structural motifs. Embo J 20: 12-18.

6. Johnson WC (1999) Analyzing protein circular dichroism spectra for accurate secondary structures. Proteins 35: 307-312.

7. Whitmore L, Wallace BA (2004) DICHROWEB, an online server for protein secondary structure analyses from circular dichroism spectroscopic data. Nucleic Acids Res 32: W668-673.

8. Whitmore L, Wallace BA (2008) Protein secondary structure analyses from circular dichroism spectroscopy: methods and reference databases. Biopolymers 89: 392-400.

9. Lees JG, Miles AJ, Wien F, Wallace BA (2006) A reference database for circular dichroism spectroscopy covering fold and secondary structure space. Bioinformatics 22: 1955-1962.

10. Sreerama N, Woody RW (2000) Estimation of protein secondary structure from circular dichroism spectra: comparison of CONTIN, SELCON, and CDSSTR methods with an expanded reference set. Anal Biochem 287: 252-260.

11. Provencher SW, Glockner J (1981) Estimation of globular protein secondary structure from circular dichroism. Biochemistry 20: 33-37.

12. Baker PR, Clauser KR http://prospector.ucsf.edu.

13. Wilkins MR, Lindskog I, Gasteiger E, Bairoch A, Sanchez JC, et al. (1997) Detailed peptide characterization using PEPTIDEMASS--a World-Wide-Web-accessible tool. Electrophoresis 18: 403-408.
