# Supplementary material for: The Disulfide Bonds in Glycoprotein E2 of Hepatitis C Virus Reveal the Tertiary Organization of the Molecule
Source: PLoS Pathog. 2010 Feb 19;6(2):e1000762. doi: 10.1371/journal.ppat.1000762 (PMC2824758; doi:10.1371/journal.ppat.1000762)
Supplement: Table S1 — Identification of disulfide bond connected HCV E2e peptides resulting from tryptic digest (0.05 MB PDF) [file ppat.1000762.s002.pdf]

Table S1: Identification of disulfide bond connected HCV E2e peptides resulting from tryptic digest

Peptide ∏	Amino acid sequence	predicted mass *	determined mass ‡
**JFH-1**			
J1	FDSSGC_{452}PGR	925,9911	n.f.
J2	LSAC_{459}R	549,6654	n.f.
J3	IGWGTLQYEDDVTNPEDMRPYC_{486}WHYPPKPC_{494}GVVPAR	4192,7303	n.f.
J4	SVC_{503}GPVYC_{508}FTPSPVVVGTTDRR	2341,7051	2341,37
J5	ADFDASTDLLC_{581}PTDC_{585}FR	1891,0769	n.f.
J6	C_{607}LVHYPYR	1051,2508	1050,63
J7	LTAAC_{644}NFTR	998,1414	998,05
	J1 + J2	1472,6515	1471,71
	J3	4190,7569	n.f.
	J4	2339,7141	2339,3
	J5	1889,0822	n.f.
	J6 + J7	2046,3921	2045,37
**UKN2b_2.8**			
U1	TALNC_{429}DDSLQTGFLASLFYVK	2307,6228	2308,00
U2	IGWGTLEYETDATNDEDMRPYC_{486}WHYPPRPC_{494}GIVSAR	4201,6514	n.f.
U3	TVC_{503}GPVYC_{508}FTPSPVVVGTTDR	2199,5445	2195,75
U4	QGVPTYSWGEDETDVFLLDSTRPPQGAWFGC_{552}TWMDGT GFTK	4586,0192	n.f.
U5	DHDSTLDLLC_{581}PTDC_{585}FR	1852,0432	1851,50
U6	C_{607}LVDYPYR	1029,1983	1028,80
U7	LNAAC_{644}DFTR	1011,1401	1010,87
	U1 + U4	6890,6802	n.f.
	U2	4199,6775	n.f.
	U3	2197,5525	2194,94
	U5	1850,0483	1849,64
	U6 + U7	2037,338	2037,59
**H77**			
H1	FDSSGC_{452}PER	998,0547	n.f.
H2	LASC_{459}R	549,6654	n.f.
H3	LTDFAQGWGPISYADGSGLDERPYC_{486}WHYPPRPC_{494}GIVP AK	4322,8613	n.f.
H4	VC_{564}GAPPC_{569}VIGGVGDNTLLC_{581}PTDC_{585}FR	2508,9457	2508,81
H5	C_{607}MVDYPYR	1047,2315	n.f.
H6	LEAAC_{644}DWTR	1065,1884	1065,98
H7	C_{652}DLEDRDR	750,8011	n.f.
H8	SELSPLLLSTTQWQVLPC_{677}SF....SSIASWAIK	6100,0588	n.f.
	H1 + H2	1544,715	1544,29
	H3	4320,8851	4321,98
	H4	2504,9486	2504,50
	H5 + H6	2109,4239	2110,35
	H7 + H8	6847,8954	6849,91

∏ Peptides were named after the isolate and numbered sequentially according to their appearance in the E2 amino acid sequence.

* Molecular mass predictions were performed using MS-BRIDGE [12] for disulfide-connected peptides and using PeptideMass for reduced peptides [13].

‡ n.f. - peptide was not found in mass spectrometry
