# Supplementary figures and images for: The Disulfide Bonds in Glycoprotein E2 of Hepatitis C Virus Reveal the Tertiary Organization of the Molecule
Source: PLoS Pathog. 2010 Feb 19;6(2):e1000762. doi: 10.1371/journal.ppat.1000762 (PMC2824758; doi:10.1371/journal.ppat.1000762)

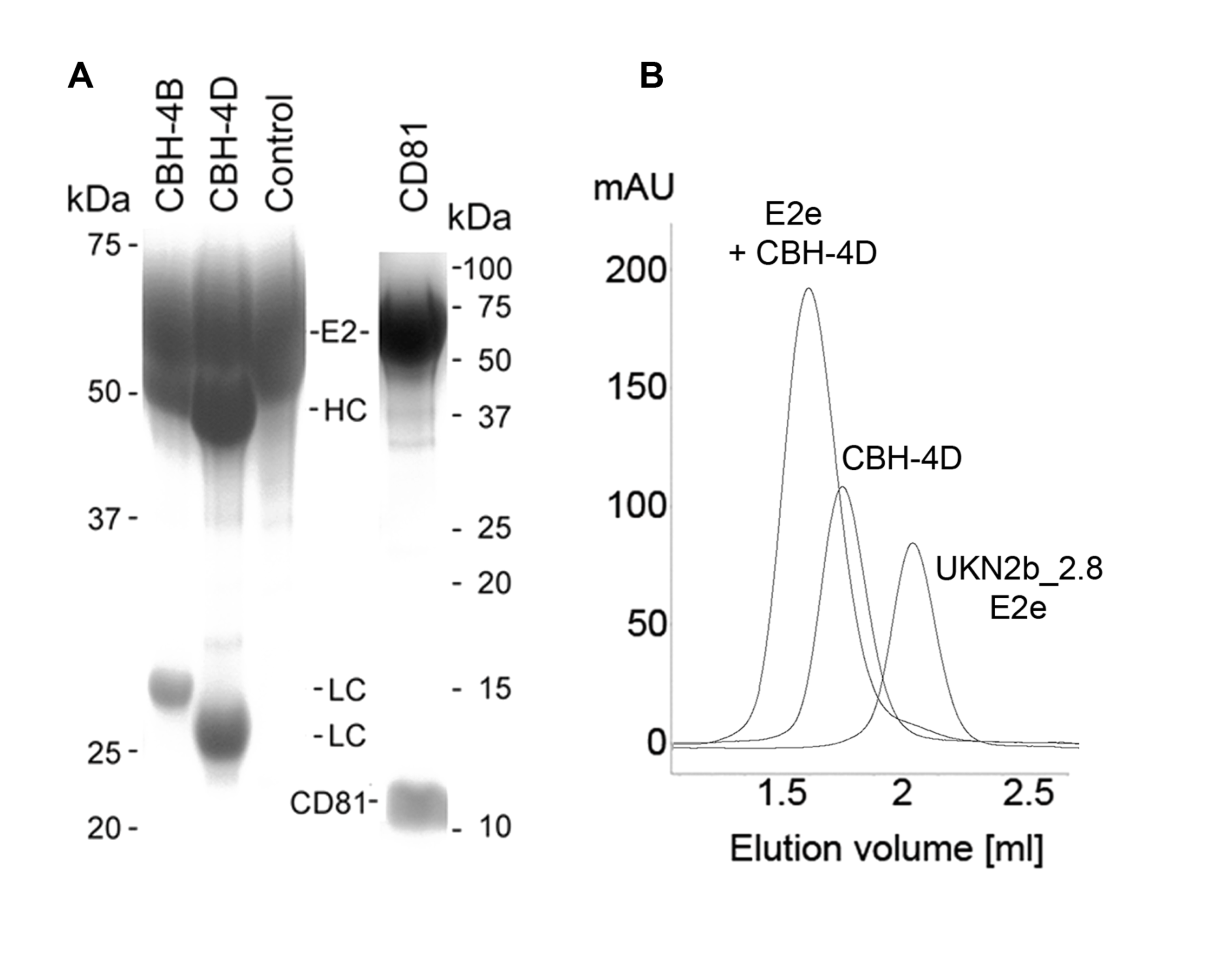

Supplement: Figure S1 — Conformational characterization of HCV E2e. A) Pull-down experiment showing that E2e specifically reacts with conformation dependent antibodies CBH-4B and CBH-4D, but not a control antibody, and binds the CD81 LEL. E2e was affinity loaded onto a Streptactin column, samples containing the respective proteins were passed through the column and the complex was eluted after washing. Elution fractions were analysed by SDS-PAGE under reducing conditions. Bands representing the E2e, CD81-LEL as well as the heavy chain (HC) and light chain (LC) of the two antibodies were observed. B) Stoichiometric complex formation between UKN2b_2.8 E2e and mAb CBH4D. UKN2b_2.8 E2e, mAb CBH4D and a mixture of the two (ratio 2:1) were loaded to the column (in three different runs) (E2e∼50kD, H53∼150kD, complex∼250kD). No peaks corresponding to either of the isolated proteins were observed in the profile of the complex, indicating a 2:1 complex stoichiometry and a high affinity of UKN2b_2.8 E2e for mAb CBH4D. (1.23 MB TIF) [file ppat.1000762.s003.tif]

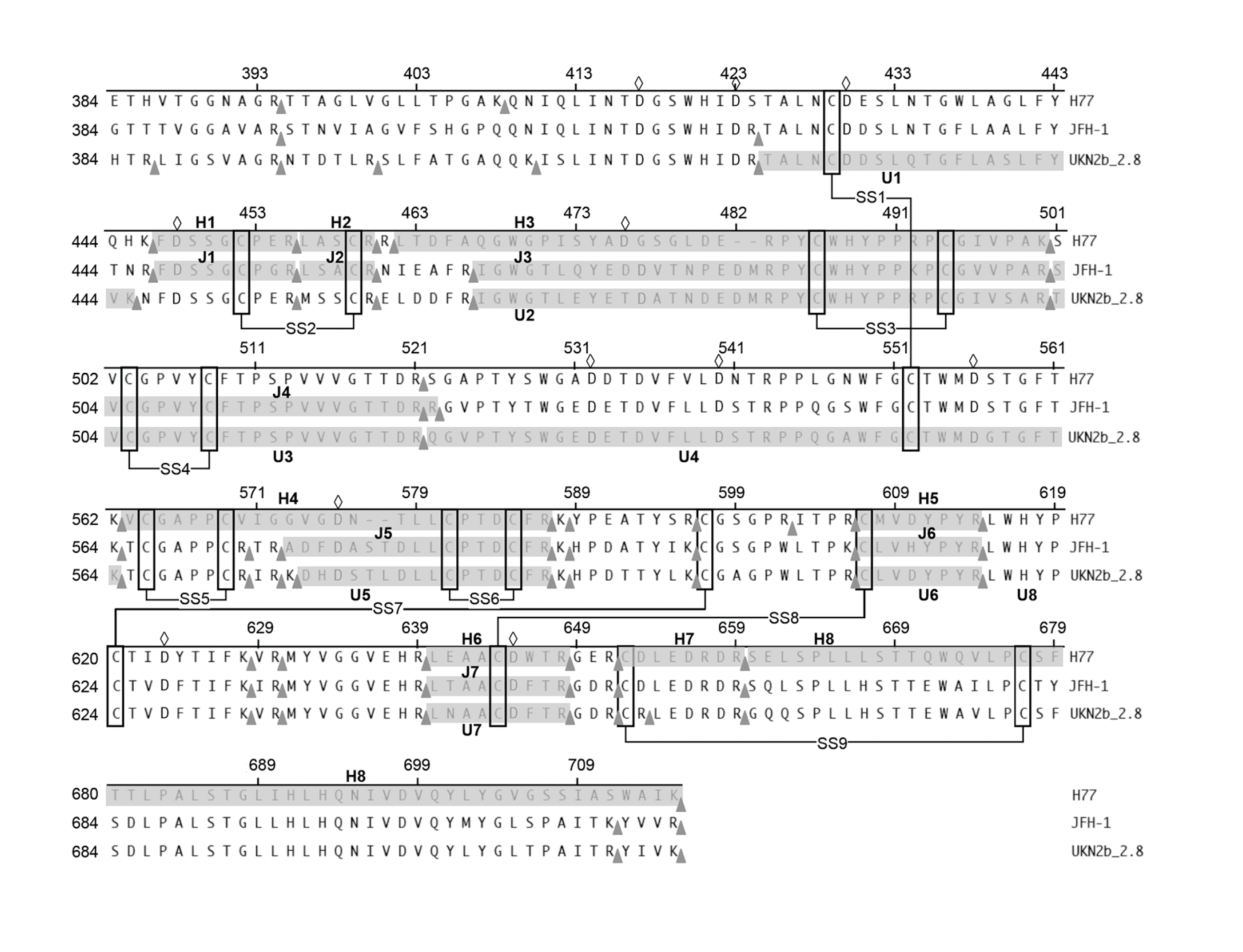

Supplement: Figure S2 — Alignment of HCV E2 amino acid sequences from strains H77, JFH-1 and UKN2b_2.8. Given the deamination of Asn residues by PNGase F, they are turned into Asp residues. Predicted trypsin cleavage sites (grey triangles) and N-glycosylation sites (empty diamonds) are indicated, cysteines are boxed and the respective disulfide bridges displayed (-SS-). Peptides identified after tryptic cleavage are shaded, named according to the respective isolate and numbered sequentially following the amino acid sequence of E2. (2.52 MB TIF) [file ppat.1000762.s004.tif]
